# Interplay of gestational parent exposure to ambient air pollution and diet characteristics on preterm birth

**DOI:** 10.1186/s12889-023-15676-x

**Published:** 2023-05-04

**Authors:** Hanna Jardel, Chantel L Martin, Cathrine Hoyo, Kristen M Rappazzo

**Affiliations:** 1 Gillings School of Global Public Health, Department of Epidemiology, University of North Carolina Chapel Hill, Chapel Hill, NC USA; 2 Oak Ridge Institute for Science and Education (ORISE) Postdoctoral Fellow at United States Environmental Protection Agency (US EPA), Research Triangle Park, NC USA; 3 Center for Environmental Health and Susceptibility, University of North Carolina Chapel Hill, Chapel Hill, NC USA; 4 Department of Biological Sciences, North Carolina State University, Raleigh, NC USA; 5 Office of Research and Development, Center for Public Health and Environmental Assessment, U.S. Environmental Protection Agency, Research Triangle Park, NC USA

**Keywords:** Pollution, Nutrition, Effect measure modification, Preterm birth

## Abstract

**Background:**

Despite many efforts, preterm birth (PTB) is poorly understood and remains a major public health problem in the United States. Toxicological work suggests gestational parent (GP) diet may modify the effect of ambient pollutants on birth outcomes. We assessed risk of PTB in humans in relation to fine particulate matter (PM_2.5_), ozone (O_3_), and nitrogen dioxide (NO_2_) and variation by diet.

**Methods:**

684 GP-singleton infant pairs in the Newborn Epigenetics Study prospective birth cohort were attributed ambient air pollutant exposures for each trimester based on residence. Total energy intake, percent of energy intake from saturated fat, and percent of energy intake from total fat were dichotomized at the 75th percentile. >We used log binomial regressions to estimate risk ratios (RR (95%CI)) for PTB by pollutant interquartile ranges, adjusting for GP age, pre-pregnancy body mass index, GP race/ethnicity, GP education, season of conception, household income, and each diet factor. We assessed departure from additivity using interaction contrast ratios (ICRs). We addressed missing covariate data with multiple imputation.

**Results:**

Point estimates suggest that O_3_ may be inversely associated with PTB when exposure occurs in trimester 2 (min RR: 0.77, 95% CI: 0.39, 1.49), but may be harmful when exposure occurs in trimester 3 (max RR: 1.51, 95% CI: 0.62, 3.64). Additionally, PM_2.5_ may be inversely associated with PTB when considered with total fat and saturated fat in trimester 2. Imprecise ICRs suggest departure from additivity (evidence of modification) with some pollutant-diet combinations.

**Conclusions:**

While confidence intervals are wide, we observed potential modification of pollutant associations by dietary factors. It is imperative that large cohorts collect the required data to examine this topic, as more power is necessary to investigate the nuances suggested by this work.

**Supplementary Information:**

The online version contains supplementary material available at 10.1186/s12889-023-15676-x.

## Introduction

Despite numerous research programs and public health initiatives, preterm birth (PTB) in the United States (US) remains a major public health challenge. In 2018, 10% of births in the US were preterm [1] and 66% of infant deaths occurred in those born preterm [2], statistics which were unchanged from the previous year [3, 4]. PTB is also associated with long-term effects. Long-term health outcomes associated with PTB include death in early adulthood (18–36 years) [5], neurodevelopmental impairment [6], use of psychiatric medications in early adulthood [7], and potentially cardiovascular disease [8]. Additionally, long-term social and economic outcomes associated with PTB include lower educational attainment [9–11], attention deficit/hyperactivity disorder [12], and collecting disability support [9, 11]. Despite decades of research, the contributing factors of PTB remain poorly understood. Gestational parent (GP) diet and environmental exposures are two potential contributors of growing interest, and may act both independently and as modifiers of adverse birth outcomes.

Among environmental exposures, ambient pollutants resulting from fossil fuel combustion are of particular interest. Many studies report positive associations between air pollutants and birth outcomes, including PTB; however, there are also studies that report null or inverse associations between air pollutant exposures and PTB, and there remain uncertainties across the research in this area, including on why some individuals may be more or less susceptible to the impacts of air pollution than others [13–15]. Air pollutants potentially act through mechanisms of inflammation and oxidative stress to increase the risk of PTB [16]. Ambient pollutants from fossil fuel combustion include, but are not limited to, particulate matter less than 2.5 microns in diameter (PM_2.5_), ozone (O_3_), and nitrogen dioxide (NO_2_), criteria air pollutants regulated under the Clean Air Act. PM_2.5_ and O_3_ levels are influenced and exacerbated by climate change due to changes in pollutant movement and reaction rates in the atmosphere [17]. Exploring factors which modify the relationship between air pollutant and birth outcomes is essential to understanding the present uncertainties in the literature and to better inform policy decisions that protect the most vulnerable.

Although GP diet has been associated with PTB, data remain inconsistent; this is in part because of differing methods of dietary analysis. Traditionally, dietary consumption has been conceptualized in research as individual food items (e.g., fish) [18]. However, individual foods are not consumed in isolation and may therefore exert differential influences on health outcomes dependent on their context. As a result, dietary consumption is increasingly conceptualized as dietary patterns (i.e., the overall diet, composed of individual food items) as opposed to consumption of individual foods; the most recent dietary guidelines published by the US federal government focused on dietary patterns [19]. GP dietary patterns, both empirically derived (e.g., exploratory factor analysis) and defined *a priori* (e.g., Western, Mediterranean, Prudent, Dietary Approach to Stop Hypertension [DASH] diets), have been investigated in relation to PTB both prior to conception and during pregnancy. This previous literature suggests that certain dietary patterns, such as those characterized by red meat, fried foods, desserts, and white bread are associated with increased risk of PTB [20, 21], while others, such as the DASH diet, Mediterranean diet, and vegetable-fruit-rice diet, are associated with decreased risk of PTB [20, 21]. The literature, however, is inconsistent [20] and faces some comparability challenges resulting from common terms being applied across various empirically-derived dietary patterns [21]. Another approach to incorporating GP diet that may help to address these issues is the use of specific nutrients as dietary indicators.

Despite the importance of understanding the interplay between ambient pollutants and diet characteristics in relation to birth outcomes, few studies focus on this topic. The four related studies focusing on ambient air pollutants (PM, NO_2_) and dietary factors (folate, fish consumption, methyl donor nutrients) in relation to various birth outcomes (PTB, livebirth, low birthweight, birth defects) [22–25] suggest associations between specific diet characteristics and ambient pollutants in relation to birth outcomes. These studies are described in more detail in the discussion. With only four studies addressing this complex topic, there is a marked paucity of research in this area.

In toxicologic research, a study in rats showed that maternal tobacco smoke effect on adverse reproductive and birth outcomes is potentially modified by maternal diet protein content [26]. Additionally, other toxicological studies of rodent models that center on overall maternal diet characteristics, such as high fat and high energy intake, provide evidence of pollutant-diet influence on later health outcomes which may indicate the potential for prenatal interactions as well [27, 28]. There has been speculation that ambient pollution and diet characteristics may together exert an influence on birth outcomes. These theories include nutrient deficiencies which hobble compensation mechanisms normally responsible for moderating physiological responses [29], potentially leading to systemic alterations including inflammation and oxidative stress [16]. Humans following diets characterized by levels of higher saturated fat have higher levels of inflammation biomarkers, lending support to these theories [30].

Despite toxicological suggestions of a diet modified effect of air pollution on birth and offspring health outcomes and tangential epidemiological work investigating diet modified effect of air pollution on birth outcomes, few epidemiologic studies have explicitly interrogated this interplay. This study investigated the association of ambient pollution on PTB and effect measure modification of this association by caloric intake, percent caloric intake from fats, and percent caloric intake from saturated fats. We hypothesize that diet characteristics, particularly saturated fat intake, will enhance any relationship between pollution and PTB.

## Methods

### Study design and population

This study utilized data from the Newborn Epigenetics Study (NEST), a prospective birth cohort in central North Carolina (NC). Pregnant individuals between 6 and 42 weeks of pregnancy (median: 15.6 interquartile range [IQR]: 11.6, 22.7 weeks) were recruited between 2009 and 2011 from prenatal clinics associated with Duke University Hospital and Durham Regional Hospital Obstetrics. Participants were required to be at least 18 years of age, to communicate in English or Spanish, and to intend delivery at one of the aforementioned hospitals. Exclusion criteria were HIV positivity, no intention to retain custody of the infant, and active plans to move residence before the infant’s first birthday.

Data on participants of the NEST cohort were gathered through interviews (English or Spanish) and medical record abstraction. Data gathered during interviews via questionnaire included sociodemographic information, occupation, medical history, lifestyle characteristics, pre-pregnancy anthropometrics, and a food frequency questionnaire (FFQ). Any questionnaire modules not completed during the interviews were sent home with participants for self-administration. During interviews, participants also contributed anthropometric measurements and biospecimens. Information abstracted from medical records included offspring information at the time of delivery including clinical estimate of gestational age, and sex. This study was approved by Institutional Review Boards at both the University of North Carolina at Chapel Hill and Duke University. In this manuscript we are choosing to use the term gestational parent instead of maternal when referring to humans in order to provide substantive specificity and to include those for which “maternal” does not apply.

### Exposure assessment

#### Air pollution

We leveraged two well-accepted daily ambient air pollution models for estimated concentrations of O_3_, PM_2.5_, and NO_2_: the EPA’s Fused Community Multiscale Air Quality (CMAQ) model [fCMAQ] [31, 32] (available at https://www.epa.gov/hesc/rsig-related-downloadable-data-files) and an ensemble model created by researchers from Harvard University [33]. The ensemble model estimates pollutant concentrations from a group of machine learning algorithms which include a neural network, random forest models, and gradient boosting models. These algorithms utilize satellite derived data (e.g., aerosol optical depth), pollutant monitoring data, meteorological data (e.g., ambient temperature, barometric pressure, wind speed), land use data (e.g., normalized difference vegetation index), elevation data, and chemical transport model predictions as inputs, and then outputs PM_2.5_ and NO_2_ concentrations at a 1 km^2^ grid. In the fCMAQ model, outputs from the Models-3/Community Multiscale Air Quality and data from the national and state level monitoring systems are combined using a bivariate Bayesian space-time downscaler approach to produce “fused” concentration estimates at the census tract level for PM_2.5_ and O_3_ [31, 32]. Ambient pollutants are represented in the metrics by which they are currently regulated: O_3_ exposure is represented as 8-hour maxima in parts per billion (ppb), PM_2.5_ as 24-hour averages in micrograms per meter cubed (µg/m^3^), and NO_2_ as 1-hour maxima in ppb.

Addresses at enrollment were geocoded by the NEST team then linked to census tract for fCMAQ output and nearest grid for ensemble model output. Concentrations for each pollutant available from each source (PM2.5 and O_3_ for fCMAQ and PM2.5 and NO2 for ensemble model) were then assigned for each day of pregnancy and averaged across trimester periods (T1, T2, T3) to produce trimester-specific estimates of air pollution exposure. PM_2.5_ daily estimates from the ensemble model were used in the primary analyses. As all air pollutant concentrations are model estimations, there is no spatial or temporal missingness in air pollution exposure assignment.

#### Diet

At enrollment, all participants were requested to complete self-administered Block FFQs for the time-period up to 6 months before pregnancy (median completion date was 134 (IQR: 119) days before delivery). The FFQ was modified to reflect dietary patterns prevalent in NC (University of Texas Anderson Cancer Center Nutrition and Lifestyle Core Questionnaire 2008v.2). This FFQ addressed frequencies and portions of intake for over 150 food items and supplements. Responses to the FFQ were analyzed by Nutrition Quest, resulting in estimates of grams per day intake of specific nutrients as well as overall daily caloric intake. Caloric intake, percent of caloric intake from saturated fat, and percent of caloric intake from total fat were used due to toxicological evidence, saturated fat being associated with inflammatory markers, and missingness in specific diet items in the FFQ. Dietary values for caloric intake, percent of caloric intake from saturated fat, and percent of caloric intake from total fat were dichotomized at the 75th percentile to designate “high” intake, using an empirical definition of “high” intake to preserve a sufficient number of records to allow models to converge. The dichotomization values for kilocalories (kcal), percent total fat, and percent saturated fat (sfat) were 2844 kcals, 35.265%, and 11.775% respectively. The dichotomization value for saturated fat, 11.775%, is marginally greater than the recommended 10% or lower percent intake from saturated fat [19]. Caloric needs change with GP characteristics and gestational period.

### Outcome assessment

We define PTB as birth before 37 weeks of completed gestation based on clinical estimate of gestational age at birth abstracted from medical records. Among those for whom clinical estimate of gestational age at birth was not available (n = 184), we recovered 23 by leveraging last menstrual period month and delivery date, applying a mean imputation with random variation. Those missing both gestational age and last menstrual period were excluded from analysis (n = 161).

### Potential confounders

Covariates considered potential confounders were determined by *a priori* assumptions encoded in a directed acyclic graph (see *Supplementary eFigure*S1) [34]. Categorical covariates included season of conception (Spring [March 21st - June 19th]; Summer [June 20th - September 21st]; Fall [September 22nd - December 20th]; Winter [December 21st - March 20th]) estimated using date of birth and gestational age, self-classified race/ethnicity (Black, non-Hispanic white, other), education (less than high school, high school or equivalent/some college, any higher degree), and annual household income while pregnant (< $10,000; $10,000 - $49,999; ≥ $50,000). Race/ethnicity was collapsed from its original categories (Black, non-Hispanic white, Hispanic, Asian/Pacific Islander, Native American, biracial, other) in order to allow our models to converge given the modest number of PTBs among the analysis sample. This variable is conceptualized as a proxy for experiences of racism, understanding that our “other” race/ethnicity category represents diverse experiences and may not always provide useful insights. Continuous variables considered were age (years) at delivery and calculated pre-pregnancy body mass index (BMI) (kg/m^3^) from self-reported pre-pregnancy height and weight. Forms of each continuous covariates were determined by a functional form analysis (linear, quadratic, restricted cubic splines, and categorical), with Akaike Information Criterion values and biological plausibility. For age and BMI variables, we collapsed the extreme 2.5% tails of the distributions (at 19 and 39 years, and 18.27 and 44.31 kg/m^2^ respectively). We used linear age and quadratic BMI in analysis models.

For sensitivity analyses, we considered dichotomous variables for eversmoking (y/n) and for having at least 30 min of outdoor exercise (jogging, walking, playing with children while walking, gardening, lawn work) per day (y/n).

### Statistical analysis

We used log binomial regression to estimate risk ratios (RRs (95%CI)) for PTB for IQR increase of each ambient air pollutant. We ran three models: model 1 included age, race, and education; model 2 included everything in model 1 as well as pre-pregnancy BMI and household income; model 3 (fully adjusted model) included everything in model 2 as well as conception season. To assess potential effect measure modification, we included interaction terms between pollutants and dietary characteristics in all models. The presence of interaction on the additive scale was determined through interaction contrast ratios (ICRs) [35, 36]. with confidence intervals calculated using the delta method [37]. Estimates for ICRs from the fully adjusted single pollutant models are considered of note if the estimate is at least 0.2 magnitude with reasonable precision (range between confidence intervals of less than 5). We addressed missing covariate data with multiple imputation using chained equations fully conditional on all other variables included in the analysis models (all exposures, outcome, all covariates, and exposure-diet characteristic interactions) with 17 iterations [38]. We aggregated analysis results using Rubin’s rules. Demographic distributions for individuals with missing gestational age were compared to the full NEST cohort.

We conducted sensitivity analyses to assess the influence of measurement error among PM_2.5_ measures by repeating analyses utilizing the fCMAQ PM_2.5_ estimates in place of the ensemble model, retaining the IQR derived from the ensemble model for RR estimates. All data processing and analysis was completed using SAS v9.4 (Cary, North Carolina) and R (Vienna, Austria) [39].

Comparing those missing gestational age (11%) to the whole cohort where non-missing covariates permit, we observed that race/ethnicity, education, and household income during pregnancy are reasonably balanced. Pre-pregnancy BMI is slightly less balanced, but this may be due to a higher proportion of missingness among those missing gestational age. The comparison between the whole NEST cohort (n = 1505) and those missing gestational age (n = 161) gives us no reason to believe that those who are missing gestational age are missing because of their gestational age: that is, we have no reason to believe that the outcome is missing not at random. This supports our use of multiple imputation to address covariate missingness.

As a sensitivity analysis, we included the dichotomous variable for ever-smoking in the fully adjusted model. As a second sensitivity analysis, we included in the fully adjusted model the dichotomous variable for outdoor exercise at least 30 min a day. In addition, we performed analyses excluding any births that did not reach the third trimester (gestational age < 189 days) to assess influence of early PTBs.

## Results

The NEST cohort is comprised of GPs largely between the ages of 25 and 29 years, majority HS graduates, a majority under/normal weight pre-pregnancy BMI, and is an overrepresentation of Black and Hispanic/other relative to the contributing population of NC and the surrounding region (Table 1). Imbalances in demographics between GPs who experienced PTB and those who did not are seen in race/ethnicity (51% Black preterm vs. 39% term), educational attainment (76% HS diploma/some college preterm vs. 68% term), household income during pregnancy (27% less than $10,000 annually preterm vs. 21% term), and pre-pregnancy BMI (27% under/normal weight preterm vs. 42% term).

**Table 1:** Study population (n = 1505) characteristics at enrollment (overall and by preterm birth)

		All participants (n = 1505)			Preterm births (n = 135)			Term births (n = 1209)			Missing gestational age (n = 161)		
		**N**	**%**		**N**	**%**		**N**	**%**		**N**	**%**	
Gestational parent age at delivery (y)													
< 20		60	3.99		2	1.48		58	4.80		--		--
25–29		742	49.30		67	49.63		673	55.67		2		1.24
30–34		347	23.06		34	25.19		312	25.81		1		0.62
35–39		169	11.23		29	21.48		140	11.58		--		--
≥ 40		28	1.86		3	2.22		25	2.07		--		--
Missing		159	10.56		--	--		1	0.08		158		98.14
Gestational parent race & Hispanic ethnicity^a^													
Black		595	39.53		69	51.11		470	38.88		56		34.78
Non-Hispanic white		422	28.04		33	24.44		357	29.53		32		19.88
Hispanic/other		454	30.17		33	24.44		382	31.60		39		24.22
Missing		34	2.26		--	--		--	--		34		21.12
Education													
No high school (HS) diploma		163	10.83		12	8.89		142	11.75		9		5.59
HS diploma/GED/some college (no degree)		1052	69.90		103	76.30		827	68.40		122		75.78
College degree		273	18.14		18	13.33		228	18.86		27		16.77
Missing		17	1.13		2	1.48		12	0.99		3		1.86
Conception season^b^													
Winter		260	17.28		28	20.74		232	19.19		--		--
Spring		304	20.20		30	22.22		274	22.66		--		--
Summer		396	26.31		37	27.41		359	29.69		--		--
Fall		384	25.51		40	29.63		344	28.45		--		--
Missing		161	10.70		--	--		--	--		161		100.00
Household income during pregnancy													
< $10,000		321	21.33		36	26.67		248	20.51		37		22.98
$10,000 - $49,999		491	32.62		44	32.59		405	33.50		42		26.09
≥ $50,000		406	26.98		34	25.19		323	26.72		49		30.43
Missing		287	19.07		21	15.56		233	19.27		33		20.50
Pre-pregnancy BMI category (kg/m^2^)													
Under/normal weight (< 25)		595	39.53		36	26.67		512	42.35		47		29.19
Overweight (25–29)		398	26.45		42	31.11		321	26.55		35		21.74
Obese (≥ 30)		455	30.23		55	40.74		363	30.02		37		22.98
Missing		57	3.79		2	1.48		13	1.08		42		26.09
Energy intake (kcal)^cd^													
Low^e^		569	37.81		55	40.74		512	42.35		2		1.24
High		175	11.63		27	20.00		148	12.24		--		--
Missing		761	50.56		53	39.26		549	45.41		159		98.76
Fat intake (%)^e^													
Low		557	37.01		59	43.70		497	41.11		1		0.62
High		187	12.43		23	17.04		163	13.48		1		0.62
Missing		761	50.56		53	39.26		549	45.41		159		98.76
Saturated fat intake (%)^f^													
Low		553	36.74		63	46.67		489	40.45		1		0.62
High		191	12.69		19	14.07		171	14.14		1		0.62
Missing		761	50.56		53	39.26		549	45.41		159		98.76

All data were from the Newborn Epigenetics Study (NEST) prospective birth cohort of individuals who delivered at Duke University Hospital or Durham Regional Hospital Obstetrics between 2009 and 2011.

a Self-classified non-Hispanic white, Black (Hispanic and non-Hispanic), other (Hispanic and non-Hispanic)

b Astronomical seasons: Spring (March 21st – June 19th ), Summer (June 20th – September 21st ), Fall (September 22nd – December 20th ), Winter (December 21st – March 20th )

c derived from food frequency questionnaire

d Kcal/day at 75th percentile (2844 kcals)

e Percent of caloric intake attributed to total fat at 75th percentile (35.265%)

f Percent of caloric intake attributed to saturated fat at 75th percentile (11.775%)

Comparing those missing diet data (50.56%) to the whole cohort (*Supplementary eTable S1)*, relative missingness in covariates is higher among those missing diet data (with absolute numbers missing similar). GP age at delivery, pre-pregnancy BMI, and season of conception are reasonably well balanced considering the difference in relative proportion of missingness between the groups. Those missing diet data had higher proportions of Black individuals, higher proportions of those with a high school diploma or equivalent, and lower proportions of those with an annual household income at least $50,000; the maximum difference in proportions between the whole cohort and those missing diet data was not greater than 8.19% points. This indicates that there may be some selection bias in the analysis sample.

Among the analysis sample, median (IQR) gestational age at enrollment was 14.4 (11.6, 21.4) weeks and at birth was 39 (38.4, 40.1) weeks. From an enrollment total of 1505, we excluded 761 without complete FFQ data, and 129 who could not be geocoded (75 missing both). We also excluded those with implausible values of daily caloric intake: those with a value of 0 kcal (n = 9) and those in the upper (5090 kcals) and lower (832 kcals) 2.5% of the kcal distribution (n = 124 and n = 136 respectively). The analysis sample was thus reduced to 684 pairs for trimester 1 and 2, and 682 for trimester 3 (pair reduction due to 2 births which occurred during trimester 2, *and thus did not experience air pollutant exposures during trimester 3*). Those missing gestational age, where data availability allows comparison, are similar to the whole cohort (Table 1). See *Supplementary eTable S2* for information about the analysis sample.

Ambient pollutants had relatively constant means and IQRs across trimesters, with the largest difference in means shown by NO_2_ at 1.11 ppb and the largest variation in IQRs seen in fCMAQ PM_2.5_ (Table 2). IQRs used in this analysis to place association estimates in context are as follows: 6 ppb NO_2_ 1-hour daily maxima, 14 ppb O_3_ 8-hour maxima; 2 µg PM_2.5_ 24-hour average. Correlations between different pollutant-trimester combinations ranged from not correlated (0.00) to highly correlated (0.97) and 46 of the 132 correlations had a magnitude of over 0.5 (*Supplementary Materials eTable 3)*. Of note, the different PM_2.5_ measures were not strongly correlated during all trimesters.

**Table 2:** Ambient pollutant exposure distributions among analysis sample

	Trimester		Mean	SD		Min	Q1	Median	Q3	Max	
**NO**_**2**_ **(ppb)** ^**a**^	**1** ^**e**^		15.92	4.55		3.51	12.87	16.04	19.65	25.02	
	**2** ^**e**^		16.71	4.32		4.55	13.96	16.66	20.49	24.60	
	**3** ^**f**^		17.03	4.49		4.68	13.91	17.39	20.73	32.96	
**O**_**3**_ **(ppb)** ^**b**^	**1**		41.90	7.93		27.71	34.16	44.49	49.01	52.19	
	**2**		40.94	8.17		26.78	32.12	42.59	48.88	53.86	
	**3**		41.52	7.68		20.54	34.08	42.76	48.91	55.61	
**Ensemble model PM**_**2.5**_ **(µg/m** ^**3**^ **)** ^**c**^	**1**		9.95	1.32		6.97	8.97	9.75	11.01	12.84	
	**2**		9.89	1.26		5.78	9.01	9.71	10.83	13.90	
	**3**		10.01	1.32		7.34	8.98	9.73	10.97	14.28	
**fCMAQ PM2.5 (µg/m** ^**3**^ **)** ^**cd**^	**1**		10.05	1.47		7.16	8.75	9.64	11.35	13.22	
	**2**		9.99	1.38		7.20	8.97	9.58	11.05	13.86	
	**3**		10.01	1.40		7.42	8.93	9.52	11.04	14.24	

Abbreviations: SD = standard deviation; min = minimum; Q1 = quantile one (25th percentile); Q3 = quantile 3 (75th percentile); max = maximum

a nitrogen dioxide (NO_2_) is reported as 1-hour daily maxima in parts per billion (ppb)

b ozone (O_3_) is reported as 8-hour maxima in ppb

c fine particulate matter (PM_2.5_) is reported as 24-hour average in micrograms (µg) per meter cubed.

d fCMAQ estimates used in sensitivity analyses

e n = 684

f n = 682

Dietary characteristics between GPs who experienced PTB and those who did not are reasonably balanced as pertains to daily energetic percent of total fat and daily energetic percent of saturated fat (Table 3). Daily caloric intake was slightly different between preterm and term, with a difference between means of 195 kcals. After dichotomizing at the 75th percentile, we again see this imbalance in caloric intake between preterm and term individuals (20% high caloric intake preterm vs. 12% term) (Table 1).

**Table 3:** Distribution of gestational parent diet characteristics (n = 684)

	Preterm^a^			Term			Total
	Mean (SD)	N		Mean (SD)	N		N
**Caloric intake (kcal/day)** ^**b**^	2530 (1283)			2334 (1081)			
** High**		50			473		523
** Low**		25			136		161
**Fat intake (%)** ^**c**^	30.49 (8.89)			30.71 (7.54)			
** High**		56			459		515
** Low**		19			150		169
**Saturated fat intake (%)** ^**d**^	9.76 (3.14)			10.18 (2.86)			
** High**		59			451		510
** Low**		16			158		174

a less than 37 weeks completed gestation

b Kcal/day at 75th percentile (2844 kcals)

c Percent of caloric intake attributed to total fat at 75th percentile (35.265%)

d Percent of caloric intake attributed to saturated fat at 75th percentile (11.775%)

The precision of pollutant estimates from the fully adjusted models is overall acceptable (max confidence limit ratio [CLR] for all models across trimesters, diet characteristics, and pollutants is 5.83). It should be noted that PM_2.5_ (both values from the ensemble model and fCMAQ) tended to be unstable, especially in second trimester models including kcal. The precision of ICR estimates in the fully adjusted model was somewhat more variable than that of the pollutant estimates, with a mean CI width of 3.71 across all models but again with PM_2.5_ models (with values from both the ensemble model and fCMAQ) offering much wider confidence intervals (up to 20.24 in the third trimester with high overall fat intake).

Pollutant estimates of note from the fully adjusted single pollutant models (all including interaction terms) (Table 4) are as follows: for the **first trimester**, PM_2.5_ seems inversely associated in models including total fat (RR (95%CI): 0.86 (0.48, 1.53)); for the **second trimester**, NO_2_ is harmful in models including sfat (RR (95%CI): 1.10 (0.75, 1.61)), O_3_ is inversely associated when considered with all diet characteristics (kcal RR (95%CI): 0.77 (0.39, 1.49); fat RR (95%CI): 0.80 (0.40, 1.64); sfat RR (95%CI): 0.79 (0.43, 1.47)), and PM_2.5_ seems to be inversely associated when considered with total fat and saturated fat (fat RR (95%CI): 0.72 (0.40, 1.30); sfat RR (95%CI): 0.77 (0.44, 1.36); for the **third trimester**NO_2_ seems inversely associated when considered with kcal and total fat (kcal RR (95%CI): 0.87 (0.57, 1.31); RR (95%CI): fat 0.83 (0.55, 1.26)), and O_3_ is harmful when considered with all diet characteristics (kcal RR (95%CI): 1.51 (0.62, 3.64); fat RR (95%CI): 1.43 (0.60, 3.38); sfat RR (95%CI): 1.36 (0.58, 3.17)).

**Table 4:** Adjusted risk ratios and 95% confidence intervals for preterm birth by interquartile range change of NO_2_, O_3_, PM_2.5_ modified by diet

	Pollutant^i^	Diet characteristics		Adjustment 1^f^ RR (95% CI)			Adjustment 2^ g^ RR (95% CI)			Adjustment 3^ h^ RR (95% CI)	
**Trimester 1**	**NO**_**2**_^**a**^	**kcal** ^**k**^		1.00	(0.69, 1.44)		1.00	(0.70, 1.43)		0.93	(0.67, 1.27)
		**fat** ^**l**^		1.09	(0.77, 1.53)		1.08	(0.76, 1.53)		0.94	(0.63, 1.39)
		**dfat** ^**m**^		1.17	(0.83, 1.63)		1.15	(0.81, 1.62)		0.99	(0.70, 1.41)
	**O**_**3**_^**b**^	**kcal**		0.91	(0.58, 1.44)		0.89	(0.56, 1.42)		0.99	(0.46, 2.12)
		**fat**		0.84	(0.55, 1.29)		0.81	(0.52, 1.26)		0.97	(0.47, 2.00)
		**sfat**		0.87	(0.58, 1.32)		0.89	(0.59, 1.33)		1.04	(0.65, 1.65)
	**H PM**_**2.5**_^** cd**^	**kcal**		0.93	(0.63, 1.37)		0.93	(0.64, 1.36)		0.94	(0.54, 1.61)
		**fat**		0.85	(0.58, 1.24)		0.84	(0.57, 1.23)		0.86	(0.48, 1.53)
		**sfat**		0.91	(0.63, 1.31)		0.91	(0.63, 1.31)		0.93	(0.57, 1.54)
	**C PM**_**2.5**_^**ce**^	**kcal**		0.97	(0.68, 1.37)		0.97	(0.69, 1.36)		1.04	(0.61, 1.75)
		**fat**		0.91	(0.64, 1.27)		0.89	(0.63, 1.26)		0.98	(0.56, 1.71)
		**sfat**		0.94	(0.68, 1.31)		0.94	(0.68, 1.31)		1.01	(0.61, 1.66)
**Trimester 2**	**NO**_**2**_	**kcal**		0.96	(0.67, 1.38)		1.00	(0.69, 1.45)		1.03	(0.69, 1.55)
		**fat**		0.95	(0.67, 1.35)		0.97	(0.68, 1.38)		0.97	(0.65, 1.44)
		**sfat**		1.05	(0.76, 1.47)		1.07	(0.77, 1.50)		1.10	(0.75, 1.61)
	**O**_**3**_	**kcal**		0.98	(0.63, 1.53)		0.95	(0.62, 1.46)		0.77	(0.39, 1.49)
		**fat**		1.12	(0.74, 1.69)		1.08	(0.71, 1.65)		0.80	(0.40, 1.64)
		**sfat**		1.03	(0.69, 1.53)		1.00	(0.67, 1.50)		0.79	(0.43, 1.47)
	**H PM**_**2.5**_	**kcal**		-- ^**j**^	--		--	--		--	--
		**fat**		0.87	(0.59, 1.28)		0.83	(0.55, 1.24)		0.72	(0.40, 1.30)
		**sfat**		0.88	(0.60, 1.29)		0.84	(0.57, 1.25)		0.77	(0.44, 1.36)
	**C PM**_**2.5**_	**kcal**		--	--		--	--		--	--
		**fat**		0.91	(0.64, 1.29)		0.88	(0.62, 1.26)		0.73	(0.40, 1.33)
		**sfat**		0.89	(0.63, 1.27)		0.87	(0.61, 1.24)		0.82	(0.48, 1.41)
**Trimester 3**	**NO**_**2**_	**kcal**		0.80	(0.57, 1.13)		0.82	(0.58, 1.16)		0.87	(0.57, 1.31)
		**fat**		0.79	(0.56, 1.11)		0.79	(0.55, 1.13)		0.83	(0.55, 1.26)
		**sfat**		0.88	(0.63, 1.23)		0.88	(0.63, 1.23)		0.95	(0.65, 1.39)
	**O**_**3**_	**kcal**		1.25	(0.77, 2.04)		1.24	(0.76, 2.01)		1.51	(0.62, 3.64)
		**fat**		1.34	(0.83, 2.16)		1.34	(0.83, 2.17)		1.43	(0.60, 3.38)
		**sfat**		1.33	(0.84, 2.12)		1.31	(0.82, 2.10)		1.36	(0.58, 3.17)
	**H PM**_**2.5**_	**kcal**		1.07	(0.71, 1.60)		1.05	(0.71, 1.54)		0.92	(0.55, 1.55)
		**fat**		1.35	(0.97, 1.89)		1.34	(0.96, 1.88)		1.07	(0.65, 1.76)
		**sfat**		1.14	(0.81, 1.63)		1.12	(0.79, 1.58)		0.95	(0.76, 1.18)
	**C PM**_**2.5**_	**kcal**		1.15	(0.78, 1.67)		1.13	(0.78, 1.64)		1.06	(0.61, 1.83)
		**fat**		1.46	(1.05, 2.02)		1.40	(0.96, 2.05)		1.21	(0.70, 2.08)
		**sfat**		1.19	(0.86, 1.65)		1.17	(0.84, 1.64)		1.00	(0.70, 1.45)

a nitrogen dioxide (NO_2_) is reported as 1-hour daily maxima in parts per billion (ppb)

b ozone (O_3_) is reported as 8-hour maxima in ppb

c fine particulate matter (PM_2.5_) is reported as 24-hour average in micrograms (µg) per meter cubed.

d Ensemble Model derived PM_2.5_ exposure

e fCMAQ derived PM_2.5_ exposure used in sensitivity analyses

f model 1: exposure + diet + exposure*diet + age + race + education

g model 2: model 1 + BMI + household income

h model 3 (fully adjusted model) : model 2 + conception season

i IQR NO_2_: 6 ppb; IQR O_3_: 14 ppb; IQR PM_2.5_: 2 µg

j “—” indicates invalid results

k Kcal/day at 75th percentile (2844 kcals)

l Percent of caloric intake attributed to total fat at 75th percentile (35.265%)

m Percent of caloric intake attributed to saturated fat at 75th percentile (11.775%)

Notable ICR results (Table 5) are only seen in the first trimester and only with sfat in models with O_3_ (ICR (95%CI) -0.35 (-2.01, 1.31)) and PM_2.5_ (ICR (95%CI)-0.29 (-2.70, 2.11)).

**Table 5:** Effect measure modification assessment of diet characteristics on fully adjusted risk ratios^f^ and 95% confidence intervals for preterm birth by interquartile range change^g^ of NO2, O3, PM2.5 modified by diet

		Caloric intake (kcal/day) ^h^		Fat intake (%) ^i^		Saturated fat intake (%) ^j^	
		interaction parameter estimate (95% CI)	ICR (95%CI)	interaction parameter estimate (95% CI)	ICR (95%CI)	interaction parameter estimate (95% CI)	ICR (95%CI)
**Trimester 1**	**NO**_**2**_^**a**^	1.20 (0.73, 1.96)	0.15 (-0.13, 0.43)	1.14 (0.64, 2.05)	0.10 (-0.27, 0.48)	0.97 (0.56, 1.69)	-0.02 (-0.54, 0.49)
	**O**_**3**_^**b**^	0.77 (0.36, 1.64)	-0.76 (-4.12, 2.60)	0.90 (0.42, 1.94)	-0.12 (-1.28, 1.03)	0.79 (0.45, 1.38)	-0.35 (-2.01, 1.31)
	**H PM**_**2.5**_^**cd**^	0.80 (0.39, 1.62)	-1.25 (-8.87, 6.38)	1.15 (0.55, 2.37)	0.13 (-0.35, 0.61)	0.85 (0.43, 1.69)	-0.29 (-2.70, 2.11)
	**C PM**_**2.5**_^**ce**^	0.81 (0.43, 1.54)	-0.77 (-5.73, 4.18)	1.03 (0.54, 1.99)	0.03 (-0.50, 0.55)	0.83 (0.44, 1.60)	-0.31 (-2.38, 1.77)
**Trimester 2**	**NO**_**2**_	1.14 (0.60, 2.16)	0.14 (-0.28, 0.55)	1.27 (0.66, 2.43)	0.13 (-0.15, 0.42)	0.86 (0.44, 1.67)	-0.17 (-1.02, 0.69)
	**O**_**3**_	1.18 (0.66, 2.12)	-0.76 (-4.12, 2.60)	0.82 (0.37, 1.84)	-0.40 (-2.91, 2.11)	1.20 (0.60, 2.41)	0.19 (-0.24, 0.62)
	**H PM**_**2.5**_	--	--	0.99 (0.48, 2.04)	-0.01 (-1.75, 1.72)	0.99 (0.53, 1.86)	0.04 (-1.17, 1.25)
	**C PM**_**2.5**_	--	--	0.86 (0.43, 1.74)	-0.50 (-4.44, 3.43)	0.96 (0.59, 1.59)	-0.03 (-1.29, 1.24)
**Trimester 3**	**NO**_**2**_	1.14 (0.62, 2.09)	0.12 (-0.49, 0.72)	1.25 (0.67, 2.30)	0.18 (-0.18, 0.54)	0.83 (0.45, 1.55)	-0.20 (-1.24, 0.84)
	**O**_**3**_	1.17 (0.51, 2.66)	0.17 (-0.51, 0.84)	0.95 (0.43, 2.08)	-0.04 (-0.35, 0.27)	1.02 (0.48, 2.15)	-0.11 (-0.58, 0.36)
	**H PM**_**2.5**_	1.36 (0.74, 2.48)	0.14 (-0.18, 0.46)	0.68 (0.33, 1.39)	-1.94 (-12.06, 8.18)	1.23 (0.84, 1.80)	0.10 (-0.08, 0.28)
	**C PM**_**2.5**_	1.24 (0.72, 2.15)	0.06 (-0.23, 0.35)	0.70 (0.36, 1.40)	-1.54 (-11.12, 8.05)	1.28 (0.75, 2.19)	0.04 (-0.32, 0.40)

a nitrogen dioxide (NO_2_) is reported as 1-hour daily maxima in parts per billion (ppb)

b ozone (O_3_) is reported as 8-hour maxima in ppb

c fine particulate matter (PM_2.5_) is reported as 24-hour average in micrograms (µg) per meter cubed.

d Ensemble Model derived PM_2.5_ exposure

e fCMAQ derived PM_2.5_ exposure used in sensitivity analyses

f model: exposure + diet + exposure*diet + age + race + education + BMI + household income + conception season

g IQR NO_2_: 6 ppb; IQR O_3_: 14 ppb; IQR PM_2.5_: 2 µg

h Kcal/day at 75th percentile

i Percent of caloric intake attributed to total fat at 75th percentile

j Percent of caloric intake attributed to saturated fat at 75th percentile

Sensitivity analyses including ever-smoking and including exercising outdoors at least 30 min per day largely failed to converge, therefore we did not include results from these analyses.

Risk ratio estimates for PM_2.5_ from the ensemble model and those from the fCMAQ were similar across all trimesters; the only discrepancies are in those involving fat characteristics in trimesters one (ensemble model PM RR (95%CI): 0.86 (0.48, 1.53) vs. fCMAQ RR (95%CI): 0.98 (0.56, 1.71)), and three (ensemble model PM RR (95%CI): 1.07 (0.65, 1.76) vs. fCMAQ PM RR (95%CI): 1.21 (0.70, 2.08)). These discrepancies are not in the direction of the estimate, but rather where the weight of the 95% CI lies on the log scale. That is, one estimate is essentially null and the other lends more support for a non-null estimate. ICR estimates from the fully adjusted models have the same signs, overall similar magnitudes, and similar widths of confidence intervals between the different PM_2.5_ values for each trimester and with each diet characteristics. The model of the first trimester PM_2.5_ exposure from fCMAQ and sfat also show an ICR of note (ICR (95%CI) -0.31 (-2.38, 1.77)), similar to the ensemble model PM_2.5_.

Results for sensitivity analyses excluding the 2 births occurring in the 2nd trimesters showed generally minor differences in effect estimates that did not impact estimate interpretation (results not shown).

## Discussion

With this analysis we investigated the effect of ambient pollution on PTB and effect measure modification by dietary characteristics. We observed complex relationships between pollutants, diet, and PTB. Specifically: point estimates for NO_2_ exposures that are harmful for PTB in trimester 2 and inversely associated in trimester 3; point estimates for O_3_ exposures that are inversely associated for PTB in trimester 2 and harmful in trimester 3; and point estimates for PM_2.5_ exposures that are inversely associated for PTB in trimesters 1 and 2. Point estimates of exposure association have 95% CIs that span the null. We observed suggestion of interaction on the additive scale in ICR values as well, though with variable ICR precision - point estimates of interaction should be considered with caution due to limited precision. More statistical power is needed to adequately investigate these complex relationships.

While our interpretations must be limited due to cohort size with complete dietary information, we did observe some evidence of effect measure modification of ambient air pollutant-PTB associations by GP dietary characteristics. The proposed mechanisms underlying this interaction include established air pollution mediated pathways of inflammation, particularly placental inflammation, systemic oxidative stress [16], and increased susceptibility to infection during pregnancy. Inflammation may impair placental function and thus may lead to fetal growth restriction, abnormal response to infection [40–42], and subsequent PTB, and certain nutrients (e.g., saturated fat) may exacerbate an inflammatory state more than others [16, 30, 43]. Oxidative stress may lead to DNA damage and subsequent cellular dysfunction or may reduce placental response to growth factors, but certain nutrients (such as methyl donor nutrients) have been associated with anti-oxidant qualities and thus may mitigate or reduce this damage [16, 25]. Of note, both inflammation and oxidative stress have been implicated in metabolic outcomes such as obesity, which has traditionally been associated with diet characteristics and is increasingly investigated in relation to ambient air pollutant exposures. There is also evidence of linkages between parent cardiometabolic conditions and PTB, and between PTB and later adverse cardiometabolic outcomes [44–46], suggesting the potential contribution of common inflammatory/oxidation pathways.

Though there have been plausible mechanisms proposed for dietary and air pollution interactions with birth outcomes, only a handful of studies examine these interactions, and few examine the same dietary factors or birth outcomes. Due to the paucity of studies evaluating associations between both ambient air pollution and diet with PTB, we are unable to directly compare the results of this study to epidemiologic studies on the same topic.

The closest related study examines folic acid supplementation before conception and PM in relation to PTB, finding interactions with all sizes of PM – those who initiated early supplemental folic acid showed a reduced impact of PM on PTB compared to those who did not initiate early folic acid supplementation [24]. Another examined folate intake and exposure to NO_2_, O_3_, PM_2.5_, and black carbon three months preconception in relation to livebirth [22]. Low folate intake modified the NO_2_ – livebirth association such that higher supplemental folic acid intake reduced the negative effects of NO_2_, but none of the other pollutant associations were strongly modified. Previous work also includes a study concerning PM_2.5_ and the frequency of fish consumption in relation to low birth weight, finding that a higher frequency of fish consumption may reduce negative impact that high PM_2.5_ has on birth weight [23]. Finally, Stingone et al. [25] examined NO_2_ methyl donor nutrient intake (including folate) specifically from food in relation to congenital heart defects, finding strong evidence of effect measure modification when considering NO_2_ in relation to perimembranous ventricular septal defect; although, the study was unable to elucidate the nuances of the complex pollutant-diet-PTB relationship due to a fairly limited analysis sample.

The results of this study taken together with the small number of related previous studies and mechanistic plausibility strengthen the case for studying ambient air pollutants and diet characteristics in relation to birth outcomes.

This question warrants further research, as it concerns both immediately modifiable factors, as well as those which may take on the scale of years to address through systematic action and technological advances [47]. It may also serve to illuminate another mechanism by which we can work to reduce the known disparities in PTB, as systemic racism makes certain populations less likely to have access to non-processed, high quality food items [48]. While the existing epidemiologic body of literature on this topic remains limited, there is a great deal of potential for connections with health care professionals and educators not only on the hazards of air pollution but also on how individual actions might change those hazards. In particular, for pregnant individuals living in areas that experience higher ambient pollution concentrations which are out of their control. Better information on and understandings of interactions between diet and environmental exposures could improve practitioner understanding and give individuals more tools to reduce environmental impacts on their health and well-being. In addition, this work adds to the body of evidence informing policy decisions around air pollutants, including those who may be more susceptible or vulnerable to the effects of air pollution.

As with nearly all studies involving live births and gestational exposure, there is potential for live birth bias. That is, fetuses exposed to stressors during gestation may be miscarried and therefore would not have been able to contribute a preterm birth outcome. This bias can lead researchers to incorrectly conclude that certain stressors result in decreased risk of preterm birth because those who survive gestation may be more resilient [49]. Previous work concerning live birth bias suggests that this may not have substantially altered results [50]. There were some noted losses in the initial NEST cohort [51], however these individuals were removed from the cohort population by design and available data does not allow us to assess the potential for bias due to fetal loss and miscarriage effectively.

This analysis assumes residential stasis over gestation. This is not an unreasonable assumption, as previous work has shown low residential mobility during gestation [52–56]. Additionally, exposure measures assume a relatively equal proportion of time indoors and outdoors for each participant, as well as relative geographic stasis when outside. This may lead to exposure mismeasurement. Diet data were not available for over 50% of the whole cohort, and comparisons of demographics between the whole cohort and those missing diet data show there may be limited selection bias. Additionally, the dietary assessment assumes a stasis in dietary status over gestation. These static representations of potentially dynamic variables may make it more difficult to isolate an association if one exists. The dietary variables also provide an extremely simple presentation of diet which, while still informative and important, is not able to reflect the nuances of the human diet and thus may obscure any influence those nuances exert. Caloric requirements vary greatly by individual and over the course of pregnancy, and these individual needs may not be ideally reflected in the available information. This analysis focuses on fat related variables because there is limited toxicological evidence supporting pollutant-diet influence on offspring health, saturated fat may contribute to inflammatory mechanisms, and specific diet items contained missingness. While this analysis was intended to be preliminary, other macronutrients are of interest beyond fat related alone. Instability in models including PM_2.5_ indicates a complex relationship which we were unable to interrogate in this analysis due to limited sample size with complete information. As such we were unable to fully assess models which included other lifestyle factors, such as smoking and physical activity, for potential influence of unmeasured confounding. While this analysis was limited in some aspects, it nevertheless has notable strengths which allow it to contribute meaningfully to the literature.

Strengths of this study include the temporal and spatial granularity in ambient pollution exposure estimates and the leveraging of detailed dietary information in conjunction with residential information, which allowed us to examine EMM of ambient air pollutants-PTB by dietary characteristics.

## Conclusions

This study should be used as a substantive contribution to the scientific literature, as well as a call to action. This understudied topic is extremely important, but the data necessary to interrogate this question are not available in larger study populations. More illuminating analyses are necessary and possible only through building larger cohorts on whom this data is collected. Additionally, future work on this topic should include consideration of exposure mixtures (of multiple exposures, and exposures at different time periods) as our participants were not exposed to only one pollutant but likely many or all pollutants simultaneously.

## Electronic supplementary material

Below is the link to the electronic supplementary material.

Supplementary Material 1

## Data Availability

The datasets generated and analyzed during the current study are not publicly available due to the presence of identifiable information on NEST participants but can be made available from Chantel L. Martin and Cathrine Hoyo upon reasonable request. Code files can be requested from corresponding author (Rappazzo.kristen@epa.gov).

## Abbreviations

BMI: Body mass index
CLR: Confidence limit ratio
CI: Confidence interval
fCMAQ: Fused Community Multiscale Air Quality
FFQ: Food frequency questionnaire
GP: Gestational parent
ICR: Interaction contrast ratio
IQR: Interquartile range
kcal: Kilocalories
kg/m^3^: Kilograms per meter cubed
NC: North Carolina
NEST: Newborn Epigenetics Study
NO_2_: Nitrogen dioxide
O_3_: ozone
PM_2.5_: Fine particulate matter (2.5 microns or smaller)
ppb: Parts per billion
PTB: Preterm birth
RR: Risk ratio
sfat: Saturated fat
T1, T2, T3: Trimester 1, 2, 3
µg/m^3^: micrograms per meter cubed
