# Supplementary material for: Interplay of gestational parent exposure to ambient air pollution and diet characteristics on preterm birth
Source: BMC Public Health. 2023 May 4;23:822. doi: 10.1186/s12889-023-15676-x (PMC10161541; doi:10.1186/s12889-023-15676-x)
Supplement: Supplementary file 1 — Supplementary Material 1 [file 12889_2023_15676_MOESM1_ESM.docx]

**Interplay of gestational parent exposure to ambient pollution and diet characteristics on preterm birth: Supplemental Materials**

eTable S1: Distributions of the overall study population compared to those missing diet data

|  |  |  | |  |  | |
| --- | --- | --- | --- | --- | --- | --- |
|  |  | **All participants**  (n = 1505) | |  | **Missing diet data** (n = 761) | |
|  |  | **N** | **%** |  | **N** | **%** |
|  |  |  |  |  |  |  |
| Gestational parent age at delivery (y) |  |  |  |  |  |  |
| < 20 |  | 60 | 3.99 |  | 38 | 4.99 |
| 25 – 29 |  | 742 | 49.30 |  | 360 | 47.31 |
| 30 – 34 |  | 347 | 23.06 |  | 127 | 16.69 |
| 35 – 39 |  | 169 | 11.23 |  | 68 | 8.94 |
| ≥ 40 |  | 28 | 1.86 |  | 9 | 1.18 |
| Missing |  | 159 | 10.56 |  | 159 | 20.89 |
| Gestational parent race & Hispanic ethnicity^a^ |  |  |  |  |  |  |
| Black |  | 595 | 39.53 |  | 339 | 44.55 |
| Non-Hispanic white |  | 422 | 28.04 |  | 152 | 19.97 |
| Hispanic/other |  | 454 | 30.17 |  | 236 | 31.01 |
| Missing |  | 34 | 2.26 |  | 34 | 4.47 |
| Education |  |  |  |  |  |  |
| No high school (HS) diploma |  | 163 | 10.83 |  | 85 | 11.17 |
| HS diploma/GED/some college (no degree) |  | 1052 | 69.90 |  | 569 | 74.77 |
| College degree |  | 273 | 18.14 |  | 99 | 13.01 |
| Missing |  | 17 | 1.13 |  | 8 | 1.05 |
| Conception season^b^ |  |  |  |  |  |  |
| Winter |  | 260 | 17.28 |  | 110 | 14.45 |
| Spring |  | 304 | 20.20 |  | 109 | 14.32 |
| Summer |  | 396 | 26.31 |  | 168 | 22.08 |
| Fall |  | 384 | 25.51 |  | 215 | 28.25 |
| Missing |  | 161 | 10.70 |  | 159 | 20.89 |
| Household income during pregnancy |  |  |  |  |  |  |
| < $10,000 |  | 321 | 21.33 |  | 185 | 24.31 |
| $10,000 - $49,999 |  | 491 | 32.62 |  | 263 | 34.56 |
| ≥ $50,000 |  | 406 | 26.98 |  | 143 | 18.79 |
| Missing |  | 287 | 19.07 |  | 170 | 22.34 |
| Pre-pregnancy BMI category (kg/m^2^) |  |  |  |  |  |  |
| Under/normal weight (< 25) |  | 595 | 39.53 |  | 263 | 34.56 |
| Overweight (25 – 29) |  | 398 | 26.45 |  | 190 | 24.97 |
| Obese (≥ 30) |  | 455 | 30.23 |  | 255 | 33.51 |
| Missing |  | 57 | 3.79 |  | 53 | 6.96 |
| All data were from the Newborn Epigenetics Study (NEST) prospective birth cohort of individuals who delivered at Duke University Hospital or Durham Regional Hospital Obstetrics between 2009 and 2011.  A Self-classified non-Hispanic white, Black (Hispanic and non-Hispanic), other (Hispanic and non-Hispanic)  b Astronomical seasons: Spring (March 21^st^ – June 19^th^), Summer (June 20^th^ – September 21^st^), Fall (September 22^nd^ – December 20^th^), Winter (December 21^st^ – March 20^th^) | | | | | | |

eTable S2: Demographic characteristics of the analysis sample by term birth

|  |  |  | |  |  | |  |  | |
| --- | --- | --- | --- | --- | --- | --- | --- | --- | --- |
|  |  | **All in analysis sample** (n = 684) | |  | **Preterm births** (n = 75) | |  | **Term births**  (n = 609) | |
|  |  | **N** | **%** |  | **N** | **%** |  | **N** | **%** |
|  |  |  |  |  |  |  |  |  |  |
| Gestational parent age at delivery (y) |  |  |  |  |  |  |  |  |  |
| < 20 |  | 20 | 2.92 |  | 2 | 2.67 |  | 18 | 2.96 |
| 25 – 29 |  | 353 | 51.61 |  | 35 | 46.67 |  | 318 | 52.22 |
| 30 – 34 |  | 202 | 29.53 |  | 21 | 28.00 |  | 181 | 29.72 |
| 35 – 39 |  | 94 | 13.74 |  | 16 | 21.33 |  | 78 | 12.81 |
| ≥ 40 |  | 15 | 2.19 |  | 1 | 1.33 |  | 14 | 2.30 |
| Missing |  | -- | -- |  | -- | -- |  | -- | -- |
| Gestational parent race & Hispanic ethnicity^a^ |  |  |  |  |  |  |  |  |  |
| Black |  | 233 | 34.06 |  | 35 | 48.00 |  | 198 | 32.35 |
| Non-Hispanic white |  | 249 | 36.40 |  | 20 | 26.67 |  | 229 | 37.77 |
| Hispanic white |  | 134 | 19.59 |  | 14 | 18.67 |  | 120 | 19.70 |
| Asian/Pacific Islander |  | 19 | 2.78 |  | 2 | 2.67 |  | 17 | 2.79 |
| Other/multiracial |  | 32 | 4.68 |  | 2 | 2.67 |  | 30 | 4.93 |
| Missing |  | 17 | 2.49 |  | 2 | 2.67 |  | 15 | 2.46 |
| Education |  |  |  |  |  |  |  |  |  |
| No high school (HS) diploma |  | 73 | 10.67 |  | 7 | 9.33 |  | 66 | 10.84 |
| HS diploma/GED/some college (no degree) |  | 446 | 65.20 |  | 57 | 76.00 |  | 389 | 63.88 |
| College degree |  | 157 | 22.95 |  | 9 | 12.00 |  | 148 | 24.30 |
| Missing |  | 8 | 1.17 |  | 2 | 2.67 |  | 6 | 0.99 |
| Conception season^b^ |  |  |  |  |  |  |  |  |  |
| Winter |  | 140 | 20.47 |  | 15 | 20.00 |  | 125 | 20.53 |
| Spring |  | 179 | 26.17 |  | 18 | 24.00 |  | 161 | 26.44 |
| Summer |  | 213 | 31.14 |  | 20 | 26.67 |  | 193 | 31.69 |
| Fall |  | 152 | 22.22 |  | 22 | 29.33 |  | 130 | 21.35 |
| Missing |  | -- | -- |  | -- | -- |  | -- | -- |
| Household income during pregnancy |  |  |  |  |  |  |  |  |  |
| < $10,000 |  | 126 | 18.42 |  | 19 | 25.33 |  | 107 | 17.57 |
| $10,000 - $49,999 |  | 207 | 30.26 |  | 23 | 30.67 |  | 184 | 30.21 |
| ≥ $50,000 |  | 242 | 35.38 |  | 20 | 26.67 |  | 222 | 36.45 |
| Missing |  | 109 | 15.94 |  | 13 | 17.33 |  | 96 | 15.76 |
| Pre-pregnancy BMI category (kg/m^2^) |  |  |  |  |  |  |  |  |  |
| Under/normal weight (< 25) |  | 304 | 44.44 |  | 23 | 30.67 |  | 281 | 46.14 |
| Overweight (25 – 29) |  | 195 | 28.51 |  | 22 | 29.33 |  | 173 | 28.41 |
| Obese (≥ 30) |  | 181 | 26.46 |  | 29 | 38.67 |  | 152 | 24.96 |
| Missing |  | 4 | 0.58 |  | 1 | 1.33 |  | 3 | 0.49 |
| Caloric intake (kcal)^cd^ |  |  |  |  |  |  |  |  |  |
| Low^e^ |  | 523 | 76.46 |  | 50 | 66.67 |  | 473 | 77.67 |
| High |  | 161 | 23.54 |  | 25 | 33.33 |  | 136 | 22.33 |
| Fat intake (%)^f^ |  |  |  |  |  |  |  |  |  |
| Low |  | 515 | 75.29 |  | 56 | 74.67 |  | 459 | 75.37 |
| High |  | 169 | 24.71 |  | 19 | 25.33 |  | 150 | 24.63 |
| Saturated fat intake (%)^f^ |  |  |  |  |  |  |  |  |  |
| Low |  | 510 | 74.56 |  | 59 | 78.67 |  | 451 | 74.06 |
| High |  | 174 | 25.44 |  | 16 | 21.33 |  | 158 | 25.94 |
|  |  |  |  |  |  |  |  |  |  |
| All data were from the Newborn Epigenetics Study (NEST) prospective birth cohort of individuals who delivered at Duke University Hospital or Durham Regional Hospital Obstetrics between 2009 and 2011.  a Self-classified race and ethnicity. Analyzed as non-Hispanic white, Black (Hispanic and non-Hispanic), other (Hispanic and non-Hispanic)  b Astronomical seasons: Spring (March 21^st^ – June 19^th^), Summer (June 20^th^ – September 21^st^), Fall (September 22^nd^ – December 20^th^), Winter (December 21^st^ – March 20^th^)  c derived from food frequency questionnaire  d daily intake  e dichotomized at the 75^th^ percentile  f percent of daily caloric intake | | | | | | | | | |

**
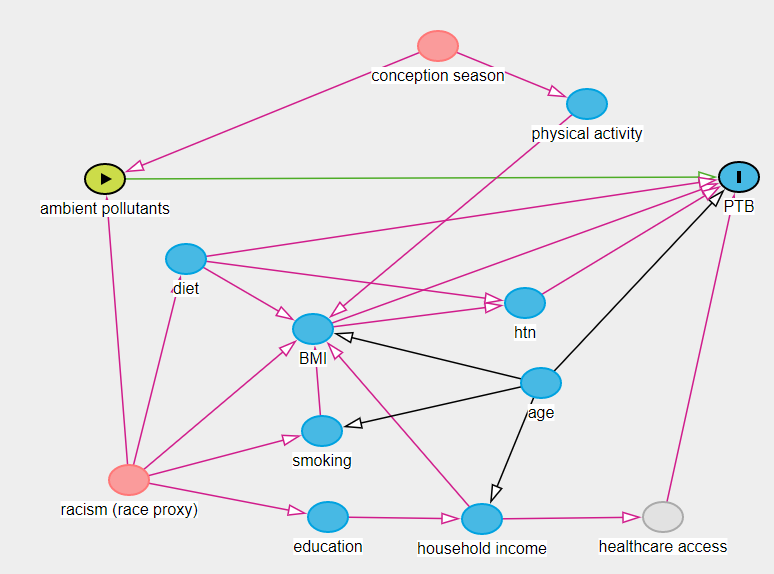
**

eFigure S1: Directed acyclic graph for this analysis

eTable S3: Correlation matrix for ambient pollutants (n = 682)

|  |  | **Trimester 1** | | | | **Trimester 2** | | | | **Trimester 3** | | | |
| --- | --- | --- | --- | --- | --- | --- | --- | --- | --- | --- | --- | --- | --- |
|  |  | **NO_2_** | **O3** | **H PM_2.5_** | **C PM_2.5_** | **NO_2_** | **O3** | **H PM_2.5_** | **C PM_2.5_** | **NO_2_** | **O3** | **H PM_2.5_** | **C PM_2.5_** |
| **Trimester 1** | **NO_2_^a^** | 1 | 0.527 | 0.109 | -0.462 | 0.301 | 0.463 | -0.320 | 0.337 | 0.586 | -0.436 | 0.248 | 0.560 |
|  | **O_3_ ^b^** | 0.527 | 1 | 0.475 | -0.279 | -0.534 | 0.184 | -0.036 | -0.250 | 0.181 | -0.104 | -0.385 | 0.076 |
|  | **H PM_2.5_^cd^** | 0.109 | 0.475 | 1 | 0.586 | -0.246 | -0.661 | 0.711 | 0.071 | -0.311 | 0.679 | -0.014 | -0.430 |
|  | **C PM_2.5_^ce^** | -0.462 | -0.279 | 0.586 | 1 | 0.013 | -0.846 | 0.818 | 0.274 | -0.675 | 0.844 | 0.292 | -0.733 |
| **Trimester 2** | **NO_2_** | 0.301 | -0.534 | -0.246 | 0.013 | 1 | -0.002 | -0.122 | 0.733 | 0.263 | -0.128 | 0.768 | 0.337 |
|  | **O_3_** | 0.463 | 0.184 | -0.661 | -0.846 | -0.002 | 1 | -0.843 | -0.134 | 0.620 | -0.882 | -0.120 | 0.666 |
|  | **H PM_2.5_** | -0.320 | -0.036 | 0.711 | 0.818 | -0.122 | -0.843 | 1 | -0.003 | -0.413 | 0.968 | -0.066 | -0.497 |
|  | **C PM_2.5_** | 0.337 | -0.250 | 0.071 | 0.274 | 0.733 | -0.134 | -0.003 | 1 | -0.044 | -0.055 | 0.955 | -0.079 |
| **Trimester 3** | **NO_2_** | 0.586 | 0.181 | -0.311 | -0.675 | 0.263 | 0.620 | -0.413 | -0.044 | 1 | -0.464 | -0.122 | 0.942 |
|  | **O_3_** | -0.436 | -0.104 | 0.679 | 0.844 | -0.128 | -0.882 | 0.968 | -0.055 | -0.464 | 1 | -0.079 | -0.528 |
|  | **H PM_2.5_** | 0.248 | -0.385 | -0.014 | 0.292 | 0.768 | -0.120 | -0.066 | 0.955 | -0.122 | -0.079 | 1 | -0.102 |
|  | **C PM_2.5_** | 0.560 | 0.076 | -0.430 | -0.733 | 0.337 | 0.666 | -0.497 | -0.079 | 0.942 | -0.528 | -0.102 | 1 |
| a nitrogen dioxide (NO_2_) is reported as 1-hour daily maxima in parts per billion (ppb)  b ozone (O_3_) is reported as 8-hour maxima in ppb  c fine particulate matter (PM_2.5_)_­_ is reported as 24-hour average in micrograms (μg) per meter cubed.  d Ensemble Model derived PM_2.5_ exposure  e fCMAQ derived PM_2.5_ exposure used in sensitivity analyses | | | | | | | | | | | | | |
